# Structural Analysis of Biodiversity

**DOI:** 10.1371/journal.pone.0009266

**Published:** 2010-02-24

**Authors:** Lawrence Sirovich, Mark Y. Stoeckle, Yu Zhang

**Affiliations:** 1 Laboratory of Applied Mathematics, Mount Sinai School of Medicine, New York, New York, United States of America; 2 Program for the Human Environment, The Rockefeller University, New York, New York, United States of America; Tata Institute of Fundamental Research, India

## Abstract

Large, recently-available genomic databases cover a wide range of life forms, suggesting opportunity for insights into genetic structure of biodiversity. In this study we refine our recently-described technique using indicator vectors to analyze and visualize nucleotide sequences. The indicator vector approach generates correlation matrices, dubbed Klee diagrams, which represent a novel way of assembling and viewing large genomic datasets. To explore its potential utility, here we apply the improved algorithm to a collection of almost 17000 DNA barcode sequences covering 12 widely-separated animal taxa, demonstrating that indicator vectors for classification gave correct assignment in all 11000 test cases. Indicator vector analysis revealed discontinuities corresponding to species- and higher-level taxonomic divisions, suggesting an efficient approach to classification of organisms from poorly-studied groups. As compared to standard distance metrics, indicator vectors preserve diagnostic character probabilities, enable automated classification of test sequences, and generate high-information density single-page displays. These results support application of indicator vectors for comparative analysis of large nucleotide data sets and raise prospect of gaining insight into broad-scale patterns in the genetic structure of biodiversity.

## Introduction

Genetic study of biodiversity has been hampered by the relatively small number of species represented in databases. For example, the largest set of alignable sequences in GenBank (small subunit ribosomal RNA) represents fewer than 21,000 species and the second largest (cytochrome b) includes fewer than 14,000 [1]. This is modest coverage compared to the approximately 1.9 million named species of plants and animals and likely much larger numbers of protozoa, fungi, bacteria, and archaea [2]. Usually, a primary goal of comparative genetic study is assembling a Tree of Life that represents the temporal sequence of evolutionary divergences. As it is computationally difficult to construct a phylogenetic tree for more than a few thousand taxa, most analyses focus on a taxonomically-restricted subset and select a few exemplars from each group (e.g., [3], [4]). Beyond computational challenges, potential limitations to tree representations include difficulty in representing discontinuities among species or groups of species, as all taxa are linked in a continuous structure; visualizing horizontal affinities across groups, as taxa within each group are joined in a single branch; and comparing data sets such as from ecological surveys, as branching diagrams challenge visual comparison.

Large, newly-available data sets [5] offer the possibility of studying genetic diversity on a wide scale. In an earlier paper, we described a method for creating “indicator vectors” representative of sets of nucleotide sequences [6]. Our aim is to develop an approach to genetic biodiversity that is computationally efficient and enables quantitative display of affinities at various taxonomic scales. Here we extend and refine this method and first apply it to large-scale differences, using sequences drawn from 12 diverse sets of animal species. On a finer scale we apply this mathematical apparatus to delineate affinities within one of the groups, North American birds, and examine biological implications of discontinuities that appear in structural representations of nucleotide sequence correlations.

### Data Preparation

We considered the 648-nucleotide region of COI employed as a standard for distinguishing animal species [5]. Inspection of terminal regions of barcode sequence alignments deposited in (BOLD) http://www.barcodinglife.org showed a high degree of ambiguous or missing nucleotides, presumably reflecting incomplete sequencing runs. To reduce this noise we restricted attention to base pair (bp) positions 100 through 600 in the downloaded alignments, a 501-nucleotide span representing 167 complete codons.

For the correlation analysis of the present framework nucleotide positions that are conserved lead to an uninformative increase in correlation, i.e., these carry no differential information. Among the 16,876 sequences of the 12 groupings considered below, we found that 161 of the 501 positions were conserved (Table 1); for the purposes of this analysis, these were dropped from analysis.

**Table 1:** Conserved sites in 501-bp sequences used for the 12-group analysis.

143	145	146	147	149	151	152	155	157	160	161	170
172	173	178	179	181	185	188	190	191	193	194	196
197	199	200	203	208	209	211	212	215	218	221	223
224	226	227	229	230	232	233	235	236	238	242	245
247	248	251	256	257	258	260	262	263	268	269	271
272	274	275	280	283	284	287	290	292	293	295	296
299	301	302	304	305	307	308	311	314	323	326	332
335	361	362	367	368	370	371	373	374	376	377	379
380	383	385	386	388	389	391	392	395	412	413	425
430	431	434	436	437	438	442	443	445	446	452	454
455	463	464	469	470	472	473	475	476	479	485	490
491	493	494	496	497	502	503	509	511	512	514	515
539	548	551	559	560	563	566	572	574	575	581	584
587	590	593	596	599							

Position 1 in table corresponds to position 5433 in mouse mitochondrial genome.

The stretch of 501 nucleotide characters can each be uniquely translated into a digital vector under the nucleotide convention as follows.
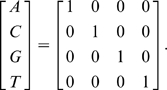
(1)


In schematic form a sequence 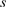 transforms to a vector 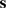 as follows
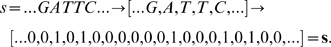
(2)


There are various metrics for calculating sequence distances based on models of nucleotide substitution. Among these the Hamming distance, 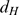, i.e., the number of substitutions required to bring two sequences of like length into agreement, is the freest of additional assumptions. More complex distances distinguish between transitions and transversions, codon positions, and equilibrium based frequencies, as for example [7]–[10]. These forms are based on evolutionary considerations, while for our approach, which is based on the present state of correlations, the Hamming distance is the metric of choice. Each COI sequence thus becomes a vector 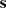 of 2004 entries; after removal of the 161 conserved nucleotides, 1,360 entries remain. The transformation of eq. (2) is not unique. An alternate transformation is
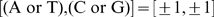
(3)which doubles, instead of quadrupling the sequence length as in eq. (2). This does not lead to the desired form of eq. (5) given below. Other alternatives that have been tried also lead to problems.

## Methods

### Distances

If two sequences of length 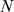 disagree at 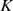 positions the Hamming distance is
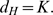
(4)


On the other hand from eq. (2) the square of its Euclidean distance 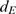 is
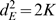
(5)and therefore
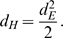
(6)


In normalized form this can be written as
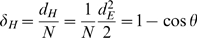
(7)which places the sequences vectors measured from a zero origin on the unit sphere and also uniquely associates the correlation coefficient 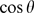, and the angle 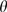, as a consequence of the law of cosines, i.e., the right hand side of eq. (7). 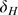 is the ratio of substitutions to site number, a customary representation.

Equations (6) & (7) are special cases of a more general recipe for associating a correlation coefficient with a metric. If 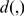 denotes a metric (distance function), then we recall that for elements 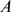, 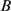 & 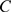 by definition the triangle inequality is satisfied
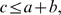
(8)where
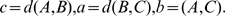
(9)


One may then show from eq. (8) that
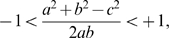
(10)which fulfills the requirement of a correlation. And if the ratio in eq. (10) is written as 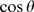 we obtain the law of cosines.
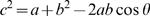
(11)


In a vector space this is exactly the case. In the construction eq. (7) 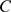 is taken as the origin.

### Indicator Vectors

For purposes of exposition consider the particular grouping of “Canadian freshwater fish” see Table 2. After the above preparation of sequence data we denote a typical fish sequence by the row vector 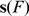. The Canadian fish dataset has 1,324 members. Next we chose 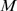 distinct sequences at random from this set and form the fish set.
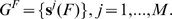
(12)


**Table 2:** COI datasets used in the 12-group analysis.

No.	BOLD Project	Group Designation	No. sequences	No. test sequences
1	GenBank-Amphibia	Amphibians	520	20
2	Barcoding of Canadian freshwater fishes	Fish	1324	824
3	Bats of Guyana	Bats	819	319
4	Birds of North America, General sequences	Birds	1688	1188
5	ACG Generalist Tachinidae	Flies	1981	1481
6	Hesperiidae of the ACG 1	Butterflies	1581	1081
7	ACG Microgastrinae	Wasps	1895	1395
8	Ants of the World, merged project	Ants	1799	1299
9	Barcoding the Aphididae	Aphids	666	166
10	GenBank-Crustacea Malac.-Decapoda	Crayfish	2068	1568
11	Marine Life, merged project	Mollusks	1652	1152
12	Genbank Cnidaria	Jellyfish	883	383
		Total	16876	10876

Datasets used to calculate and test group indicator vectors.

In general if there are 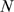 groupings we consider 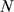 sets 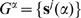, where 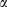 ranges over the 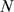 groupings.

An indicator unit vector 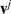 for each set is then determined on the basis that it have a maximal correlation with the selected 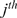 taxon, and minimal correlation with all other taxa [6]. As a simple but useful illustration consider 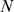 sequence vectors 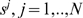, say one representative from each of 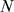 groups, or each an average of each group. We then seek 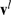, the 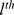 indicator vector such that
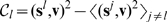
(13)is a maximum,
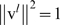
(14)where 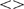 signifies the average. It is straightforward to show that under the reasonable assumption that if 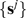 are linearly independent then the criterion function 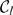 has a positive maximum and that it is determined as the eigenvector with the largest (positive) eigenvalue of
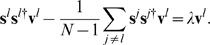
(15)


One consequence of the particular criterion for choosing the 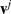 is that it provides a natural structural representation expressed as auto- and cross-correlations, given by
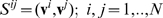
(16)and referred to as the structure matrix. We also define the diversity matrix as given by
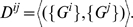
(17)


This notation denotes the mean over all 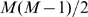 inner products pairs of the members of 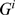 with those of 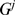, which thus gives a depiction of within and among group correlations.

A fixed number of members, 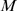, in the sets 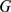 confers equal weights on each of the taxa. These may be considered as the “training set,” for the indicator vector and the remaining sequences are used as a “test set.” There is reason to make 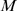 relatively small in initial calculations. Once past the testing stage there may be reason to take 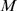 as large as possible within the restriction of equal weightings.

### Probabilities

Another consequence of embedding a character sequence into a vector space, eq. (2), is that the average of an ensemble of sequences 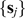 can be defined as
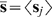
(18)Which through the inverse operation of eq. (2) furnishes the probability of occurrence of (A,T,C,G) at each nucleotide position and that
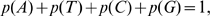
(19)which is a consequence of eq. (2).

#### Conservation of Probability

Eq. (4) allows us to regard the 4-vectors as specifying the probabilities of the associated symbols. We now demonstrate that this property is inherited by the indicator vectors, i.e., its 4-vectors sum to unity. To see this define
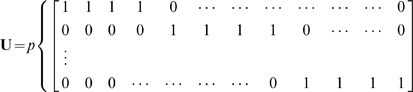
(20)where 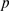, the number of rows, is also the number of bps. Multiplication of (15) by 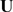 yields
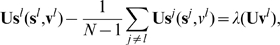
(21)but
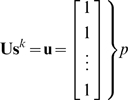
(22)for any 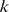 and from this it follows that
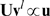
(23)which proves the assertion. (This proof depends specifically on regarding an unknown bp as 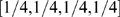, which we deem to be reasonable.) Therefore each indicator vector can be regarded as 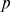 quartets of probability in the four possible symbols.

### Tree Construction

A customary practice is to express sequence separations as distances, which play a role in the construction of trees. It is straightforward to show the connection of distances to the correlations contained in eq. (16) and of eq. (17). In fact it directly follows from eq. (7) that
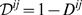
(24)is the matrix of average Hamming distances between taxons 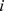 and 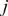. By the same token
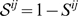
(25)is the distance matrix between the 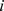 & 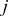 indicator vectors. It is important to note that evolutionary considerations do not figure in the calculation of the above distances.

## Results

We first considered 12 animal groups, using COI sequences deposited in BOLD taxon-specific projects (Table 2). In all cases analysis was restricted to sequences of sufficient length, and excluded those containing excessive blank positions.

**Figure 1:**
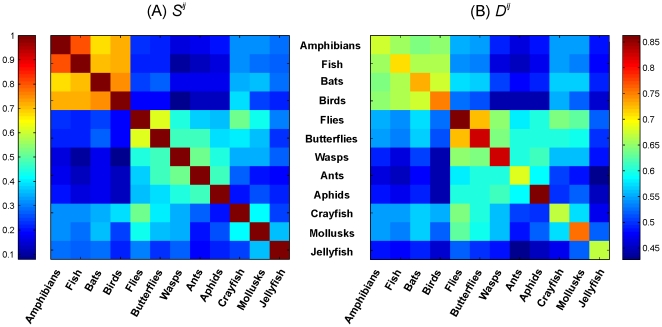
Correlations among indicator vectors for 12 animal groups. (A) Structure matrix eq. (16). (B) Diversity matrix eq. (17). Numerical forms of matrices given in Table 3. Differing color bar scales in (A) and (B) are used to emphasize off diagonal resemblance between matrices.

The structure matrix for the 12 groups displays correlations among their respective indicator vectors (Figure 1A). These are arranged in large-scale taxonomic divisions [Chordata, Arthropoda (Insecta, Malacostraca), Mollusca, Cnidaria], and sub-ordered based on correlations, e.g., within the upper 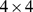 matrix (Chordata), groups are ordered by vector correlation as quantified by
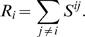
(26)


Thus amphibians have the highest relationships with the others in this set. The next 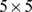 block representing Class Insecta, are ordered by relationship as above. The diversity matrix eq. (17) quantifies the degree of diversity within and among data sets (Figure 1B), and has an impressionistic similarity to the structure matrix of unitary indicator vectors (Figure 1A). The diagonal elements of Figure 1B illustrate the high internal diversity of amphibians, ants, crayfish, and jellyfish, and relative lack of internal diversity for flies, butterflies, wasps, and aphids. Numerical equivalents of Figure 1 are given in Table 3. Lack of diversity might be consistent with these data being drawn from single families or subfamilies. Diversity as defined by (24) introduces an objective measure of diversity based on variance.

**Table 3:** Numerical representations of Figure 1A and 1B, respectively.

**(A)**											
1.0000	0.8060	0.6799	0.7221	0.2215	0.2201	0.1594	0.1754	0.1920	0.3220	0.3252	0.2894
0.8060	1.0000	0.7146	0.7260	0.2169	0.2209	0.1021	0.1432	0.1587	0.3199	0.2898	0.2706
0.6799	0.7146	1.0000	0.7458	0.2477	0.2662	0.1729	0.1792	0.1754	0.3498	0.3565	0.2872
0.7221	0.7260	0.7458	1.0000	0.1900	0.1806	0.0787	0.1372	0.1284	0.3841	0.2514	0.2225
0.2215	0.2169	0.2477	0.1900	1.0000	0.6219	0.4446	0.3790	0.3526	0.5245	0.4358	0.3034
0.2201	0.2209	0.2662	0.1806	0.6219	1.0000	0.4708	0.4775	0.3794	0.4120	0.3648	0.2397
0.1594	0.1021	0.1729	0.0787	0.4446	0.4708	1.0000	0.5160	0.4222	0.3455	0.3363	0.2616
0.1754	0.1432	0.1792	0.1372	0.3790	0.4775	0.5160	1.0000	0.4753	0.2803	0.2062	0.1844
0.1920	0.1587	0.1754	0.1284	0.3526	0.3794	0.4222	0.4753	1.0000	0.1980	0.2540	0.2043
0.3220	0.3199	0.3498	0.3841	0.5245	0.4120	0.3455	0.2803	0.1980	1.0000	0.4127	0.2409
0.3252	0.2898	0.3565	0.2514	0.4358	0.3648	0.3363	0.2062	0.2540	0.4127	1.0000	0.3756
0.2894	0.2706	0.2872	0.2225	0.3034	0.2397	0.2616	0.1844	0.2043	0.2409	0.3756	1.0000
**(B)**											
0.6826	0.6633	0.6545	0.6567	0.5603	0.5500	0.4977	0.4793	0.4885	0.5547	0.5577	0.4916
0.6633	0.7144	0.6686	0.6715	0.5464	0.5413	0.4682	0.4611	0.4656	0.5494	0.5447	0.4842
0.6545	0.6686	0.7433	0.6877	0.5783	0.5731	0.5055	0.4905	0.4877	0.5726	0.5767	0.5031
0.6567	0.6715	0.6877	0.7600	0.5295	0.5191	0.4453	0.4527	0.4433	0.5616	0.5279	0.4680
0.5603	0.5464	0.5783	0.5295	0.8623	0.7314	0.6482	0.5874	0.5971	0.6521	0.6366	0.5323
0.5500	0.5413	0.5731	0.5191	0.7314	0.8306	0.6480	0.6083	0.6000	0.6148	0.6034	0.5021
0.4977	0.4682	0.5055	0.4453	0.6482	0.6480	0.8185	0.6034	0.6025	0.5632	0.5663	0.4897
0.4793	0.4611	0.4905	0.4527	0.5874	0.6083	0.6034	0.6920	0.5857	0.5198	0.4936	0.4360
0.4885	0.4656	0.4877	0.4433	0.5971	0.6000	0.6025	0.5857	0.8671	0.5039	0.5144	0.4487
0.5547	0.5494	0.5726	0.5616	0.6521	0.6148	0.5632	0.5198	0.5039	0.6820	0.5828	0.4826
0.5577	0.5447	0.5767	0.5279	0.6366	0.6034	0.5663	0.4936	0.5144	0.5828	0.7585	0.5286
0.4916	0.4842	0.5031	0.4680	0.5323	0.5021	0.4897	0.4360	0.4487	0.4826	0.5286	0.6680

**Figure 2:**
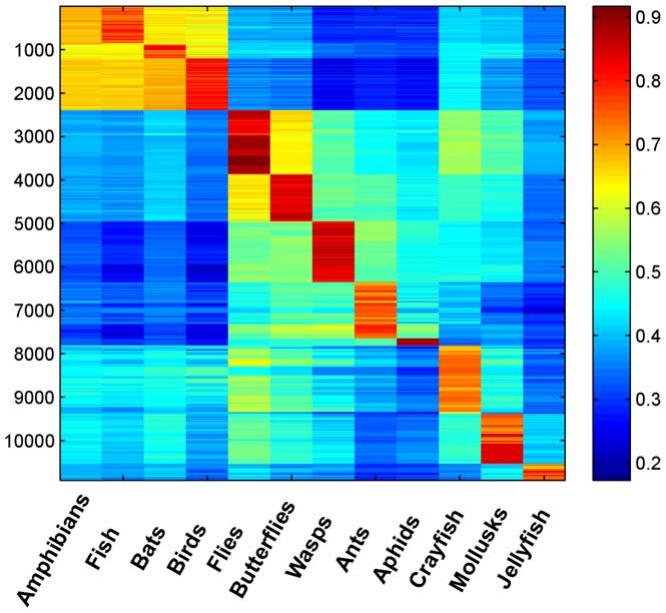
Prediction matrix with 10,876 individual sequence vectors (rows) applied to 12 group-level indicator vectors (columns). Test sequences are arranged to follow order of indicator vectors, such that blocks of high correlation near diagonal represent affinity with their respective group vector. Available test sequences ranged from 20 (amphibians) to 1,568 (crayfish), thus generating blocks of varying sizes as shown.

We applied the 12 indicator vectors to the remaining set of 10,876 test sequences, generating a structure matrix of correlations, (Figure 2). With one interesting set of exceptions, there were no assignment errors, i.e., each individual test sequence was most highly correlated with its respective group-level vector. The exceptions were 33 sequences, .09% of all sequences, in the fish dataset which, according to the metric, were more closely correlated with the amphibian than the fish indicator vector. Inspection revealed that each error was caused by a lamprey (Class Cephalospidomorphi) sequence and all lamprey sequences produced this erroneous assignment. The remaining sequences in the Canadian fish dataset represented ray-finned fishes (Class Actinopterygii). Viewed taxonomically, the lampreys appear to be inadvertently included in fish dataset; when removed there was 100% accuracy of assignment of test sequences plus training sequences.

**Figure 3:**
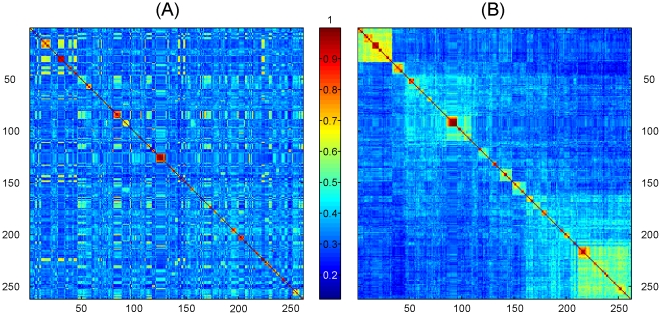
Correlations among indicator vectors for 262 species of North American birds. (A) Species alphabetically ordered by genus. (B) Species ordered by established taxonomic order [11].

**Figure 4:**
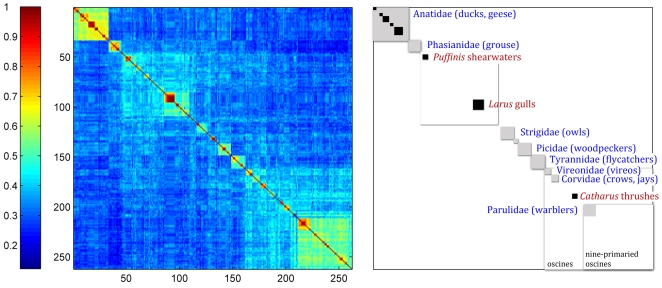
Annotated structure matrix of 262 North American bird species arranged in taxonomic order reflecting phylogenetic relationships. Representational fractures define “boxes” which correspond to taxonomic divisions.

We applied the indicator vector approach at a finer scale, analyzing differences within the dataset of North American birds, which contained 1,693 sequences representing 558 species. As a compromise between a large 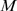 and a large test set, we chose 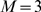, giving 262 admissible species and 471 test sequences. With the input ordered alphabetically by taxonomic genus, the resulting structure matrix appears to be disordered with small regions of high correlation (Figure 3A). When arranged in a taxonomic order representing phylogenetic relationships [11] (Table 4), these correlations coalesced into a coherent picture (Figure 3B), which could be viewed as taxonomy organizing the structure matrix according to closeness of correlations. Discontinuities in the correlation among North American birds, evident as “boxes” or “blocks” in the color matrix, corresponded to avian taxonomic divisions (Figure 4). Most of the blocks represented families, with some blocks corresponding to lower (genera) or higher (suborder) groupings (Figure 4).

**Table 4:** List of North American bird species arranged according to AOU Check-list(2009).

1	Anser albifrons	67	Pandion haliaetus	133	Selasphorus rufus	199	Catharus fuscescens
2	Chen caerulescens	68	Accipiter striatus	134	Megaceryle torquata	200	Catharus bicknelli
3	Branta bernicla	69	Accipiter cooperii	135	Megaceryle alcyon	201	Catharus ustulatus
4	Branta hutchinsii	70	Accipiter gentilis	136	Chloroceryle americana	202	Catharus guttatus PS-1
5	Branta canadensis	71	Buteo swainsoni	137	Melanerpes lewis	203	Catharus guttatus PS-2
6	Aix sponsa	72	Falco columbarius	138	Melanerpes formicivorus	204	Hylocichla mustelina
7	Anas strepera	73	Gallinula chloropus	139	Melanerpes carolinus	205	Oreoscoptes montanus
8	Anas americana	74	Fulica americana	140	Sphyrapicus thyroideus	206	Toxostoma rufum
9	Anas rubripes	75	Grus americana	141	Sphyrapicus varius	207	Sturnus vulgaris
10	Anas platyrhynchos	76	Pluvialis dominica	142	Sphyrapicus nuchalis	208	Motacilla tschutschensis
11	Anas discors	77	Charadrius semipalmatus	143	Sphyrapicus ruber	209	Motacilla alba
12	Anas clypeata	78	Charadrius melodus	144	Picoides nuttallii	210	Bombycilla cedrorum
13	Anas acuta	79	Haematopus bachmani	145	Picoides villosus	211	Peucedramus taeniatus
14	Anas carolinensis	80	Actitis macularius	146	Picoides albolarvatus	212	Parula americana
15	Aythya valisineria	81	Tringa glareola	147	Picoides dorsalis	213	Dendroica caerulescens
16	Aythya americana	82	Limnodromus griseus	148	Colaptes auratus	214	Dendroica coronata
17	Aythya collaris	83	Gallinago delicata	149	Contopus sordidulus	215	Dendroica nigrescens
18	Aythya fuligula	84	Scolopax minor	150	Empidonax flaviventris	216	Dendroica townsendi
19	Aythya marila	85	Phalaropus lobatus	151	Empidonax alnorum	217	Dendroica occidentalis
20	Aythya affinis	86	Rissa tridactyla	152	Empidonax traillii	218	Dendroica graciae
21	Somateria fischeri	87	Larus ridibundus	153	Empidonax minimus	219	Dendroica pinus
22	Somateria spectabilis	88	Larus atricilla	154	Empidonax hammondii	220	Protonotaria citrea
23	Somateria mollissima	89	Larus heermanni	155	Empidonax difficilis	221	Seiurus aurocapilla
24	Histrionicus histrionicus	90	Larus canus	156	Pyrocephalus rubinus	222	Oporornis philadelphia
25	Melanitta fusca	91	Larus occidentalis	157	Myiarchus tuberculifer	223	Geothlypis trichas
26	Melanitta nigra	92	Larus californicus	158	Myiarchus cinerascens	224	Piranga rubra
27	Clangula hyemalis	93	Larus smithsonianus	159	Myiarchus tyrannulus	225	Pipilo erythrophthalmus
28	Bucephala albeola	94	Larus fuscus	160	Pitangus sulphuratus	226	Aimophila cassinii
29	Bucephala clangula	95	Larus glaucescens	161	Myiodynastes luteiventris	227	Spizella pallida
30	Bucephala islandica	96	Onychoprion aleuticus	162	Lanius ludovicianus	228	Spizella breweri
31	Lophodytes cucullatus	97	Thalasseus maximus	163	Vireo griseus	229	Spizella pusilla
32	Mergus merganser	98	Thalasseus sandvicensis	164	Vireo solitarius	230	Amphispiza bilineata
33	Mergus serrator	99	Thalasseus elegans	165	Vireo huttoni	231	Amphispiza belli
34	Perdix perdix	100	Stercorarius pomarinus	166	Vireo philadelphicus	232	Calamospiza melanocorys
35	Bonasa umbellus	101	Stercorarius parasiticus	167	Vireo olivaceus	233	Passerculus sandwichensis
36	Centrocercus urophasianus	102	Stercorarius longicaudus	168	Vireo flavoviridis	234	Passerella iliaca
37	Falcipennis canadensis	103	Uria aalge	169	Cyanocitta cristata	235	Melospiza lincolnii
38	Lagopus lagopus	104	Alca torda	170	Aphelocoma californica PS-1	236	Melospiza georgiana
39	Lagopus muta	105	Cepphus grylle	171	Gymnorhinus cyanocephalus	237	Zonotrichia albicollis
40	Lagopus leucura	106	Brachyramphus marmoratus	172	Nucifraga columbiana	238	Zonotrichia atricapilla
41	Dendragapus obscurus	107	Brachyramphus brevirostris	173	Pica nuttalli	239	Junco hyemalis
42	Tympanuchus phasianellus	108	Cerorhinca monocerata	174	Corvus caurinus	240	Junco phaeonotus
43	Tympanuchus pallidicinctus	109	Fratercula arctica	175	Corvus corax PS-1	241	Calcarius mccownii
44	Meleagris gallopavo	110	Zenaida macroura	176	Tachycineta bicolor	242	Calcarius ornatus
45	Oreortyx pictus	111	Columbina inca	177	Poecile gambeli PS-1	243	Cardinalis cardinalis
46	Gavia pacifica	112	Columbina passerina	178	Poecile gambeli PS-2	244	Pheucticus melanocephalus
47	Gavia adamsii	113	Myiopsitta monachus	179	Poecile sclateri	245	Passerina amoena
48	Podiceps grisegena	114	Coccyzus erythropthalmus	180	Poecile rufescens	246	Passerina versicolor
49	Fulmarus glacialis PS-1	115	Crotophaga ani	181	Poecile cincta	247	Passerina ciris
50	Puffinus creatopus	116	Tyto alba	182	Auriparus flaviceps	248	Dolichonyx oryzivorus
51	Puffinus carneipes	117	Megascops kennicottii PS-1	183	Sitta canadensis	249	Agelaius phoeniceus
52	Puffinus pacificus	118	Megascops kennicottii PS-2	184	Sitta carolinensis	250	Xanthocephalus xanthocephalus
53	Puffinus bulleri	119	Megascops asio	185	Sitta pygmaea	251	Euphagus cyanocephalus
54	Puffinus tenuirostris	120	Bubo virginianus	186	Campylorhynchus brunneicapillus	252	Quiscalus major
55	Oceanodroma leucorhoa	121	Strix occidentalis	187	Salpinctes obsoletus	253	Quiscalus mexicanus
56	Morus bassanus	122	Strix varia	188	Thryothorus ludovicianus	254	Molothrus aeneus
57	Phalacrocorax penicillatus	123	Strix nebulosa	189	Thryomanes bewickii PS-1	255	Icterus cucullatus
58	Phalacrocorax carbo	124	Asio otus	190	Cinclus mexicanus	256	Icterus bullockii
59	Phalacrocorax pelagicus	125	Asio flammeus	191	Regulus satrapa	257	Icterus gularis
60	Ardea herodias	126	Aegolius acadicus	192	Regulus calendula	258	Leucosticte tephrocotis
61	Ardea alba	127	Nyctidromus albicollis	193	Polioptila caerulea	259	Carpodacus cassinii
62	Egretta tricolor	128	Phalaenoptilus nuttallii	194	Luscinia svecica	260	Carpodacus mexicanus
63	Bubulcus ibis	129	Chaetura vauxi	195	Sialia sialis	261	Carduelis hornemanni
64	Eudocimus albus	130	Archilochus colubris	196	Sialia mexicana	262	Passer domesticus
65	Plegadis chihi	131	Stellula calliope	197	Sialia currucoides		
66	Coragyps atratus	132	Selasphorus platycercus	198	Myadestes townsendi		

Among the 471 test bird sequences, there were 16 apparently incorrect assignments distributed among 4 species pairs (*Junco phaneotus/J. hyemalis; Anas platyrhynchos/A. rubripes; Larus smithsonianus/L. glaucescens; Sphyrapicus ruber/S. nuchalis*). In the first instance each sequence set of 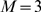 were identical so that the indicator vectors were also identical. In the remaining cases the indicator vectors were close but not equal reflecting the fact that the defining sequence sets shared some identical members. While such singular behavior is revealed by the present algorithm, these sets of species were previously noted to be indistinguishable by COI barcode [12].

**Figure 5:**
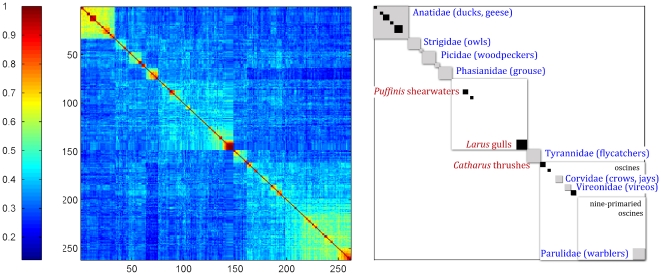
Annotated structure matrix of 262 North American bird species according to NJ tree ranking.

**Figure 6:**
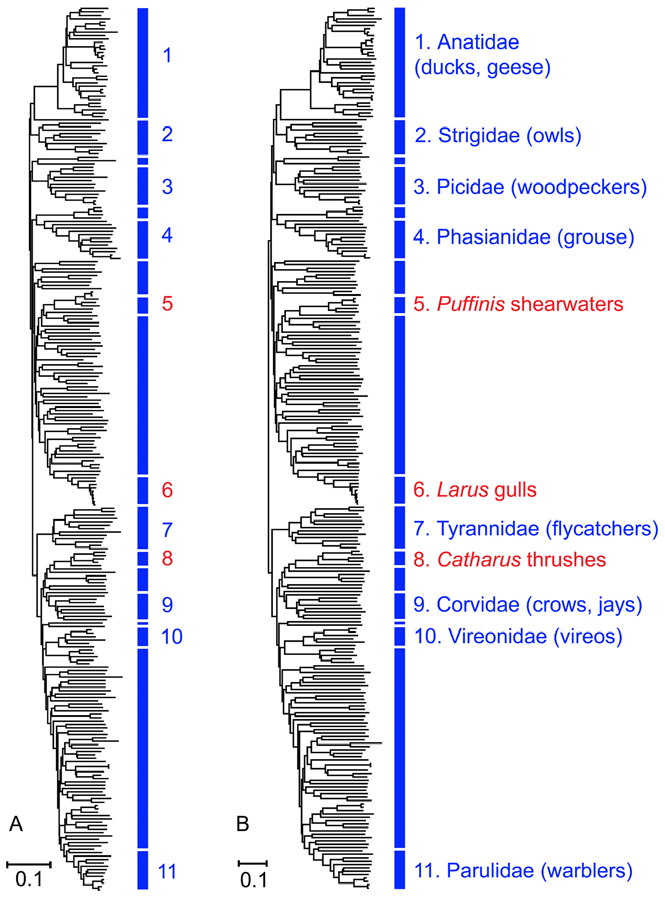
Comparison of NJ trees based on the structure matrix right, and on the diversity matrix, left.

As indicated in eq. (24) the structure matrix 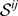 can be directly associated with a matrix of inter-species distances 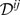. Since such a matrix can be made the basis of tree constructions we can apply the neighbor joining (NJ) algorithm of Saitou and Nei [13] to 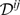. Using consistency arguments [14], [15], Bryant [16] has demonstrated that the NJ construction is a unique clustering algorithm of the distance matrix [17]. Since the distance matrix is based on genomic distances, and not on evolutionary hypotheses, we can view the resulting NJ tree as intrinsic to the data. The species ordering according to this tree produces the structure matrix shown in Figure 5. This demonstrated the same set of clusters as seen in Figure 4; only the order of clusters differed. Thus at this level of resolution the indicator vector approach to classification coupled with NJ provides a self-generating ranking that is in general agreement with established taxonomy. Figure 6 compares the NJ tree that emerges from the structure matrix with the tree that derives from the averaged Hamming distance matrix between species, is equivalent to the diversity matrix (17).

## Discussion

**Figure 7:**
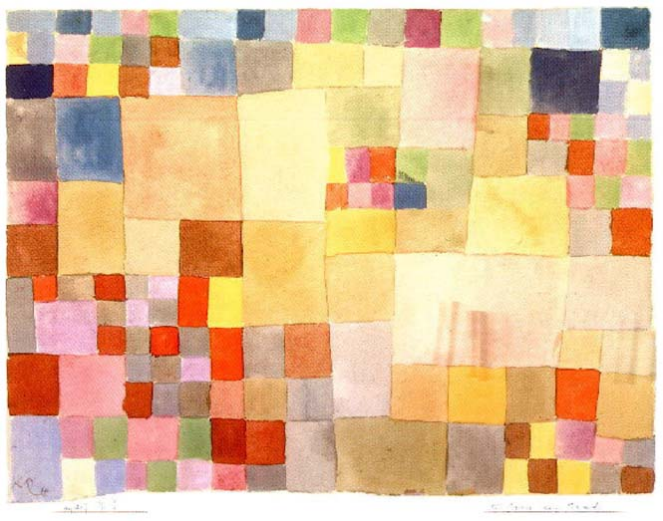
Flora on Sand by Paul Klee.

This paper describes a mathematical approach to comparative analysis of nucleotide sequences using digital transformation in vector space. We term the resulting structure matrices “Klee diagrams”, in acknowledgement of the geometric paintings of artist Paul Klee (see Figure 7). This approach is of general utility and could be applied to any set of aligned sequences. In this study we explore its potential by analyzing a large, diverse set of DNA barcodes, the short segment of mitochondrial COI gene employed as a standard for identification of animal species (6). The resulting Klee diagrams display the structure of present-day mitochondrial genetic diversity, a “macroscopic” view of the products of evolution [18], [19]. This approach is akin to a distance metric (see Methods), and in fact the matrix of indicator vector correlations can be used to generate an NJ tree (Figure 6).

**Figure 8:**
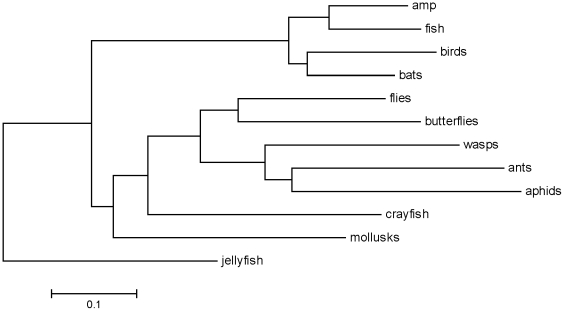
The NJ tree corresponding to the structure matrix depicted in Figure 2.

As compared to standard distance metrics with neighbor-joining, indicator vectors preserve character probabilities that distinguish sequence sets, enable automated classification of test sequences, and generate high-information density displays without constraints of tree diagrams. Regarding the latter point, as one example, the 12-group Klee diagram displays affinity among flies and crayfish, a finding which might be of interest for further exploration, and yet this sort of horizontal similarity is not represented in the NJ tree diagram, shown in Figure 8. Discontinuities in indicator vector correlations, evident as blocks in Klee diagrams, corresponded to branches in the tree; for example, in North American bird matrix, these blocks represent families, genera, and sub-orders (Figures 4, 5). These results, generated with a small sample of world birds, suggest that this approach might be usefully applied to generate a classification for poorly-studied groups by combining DNA barcodes with indicator vector analysis. Such a classification could be refined when additional morphologic, ecological, and genetic study was available.

The results so far suggest natural discontinuities, or fractures, in the genetic structure of biodiversity, at least as reflected in animal mitochondrial genomes. In quantitative terms, blocks represent higher correlation within than among sets of sequences. Further study will help determine the nature of underlying mitochondrial differences, for instance whether species- and family-level blocks, for example, reflect differences in coding or non-coding positions. The present-day discontinuities seen in Klee diagrams may not be evident from a historical perspective, such as in a phylogenetic tree which links all forms in a continuous structure. It is of interest to reconcile these two perspectives, namely the continuous nature of evolution with the fractures in present-day genetic biodiversity; these might be viewed, respectively, as “time-like” and “space-like”. One may speculate on the relation of such jump phenomena to adaptive radiations and the punctuated equilibrium model of evolution [20]. It may be possible to make useful observations for time-like behavior from space-like behavior as was done through the ergodic theory of statistical physics, [21].

As currently developed, our approach is limited to complete sets of homologous sequences, rather than overlapping sets of incomplete data as are often used in phylogenetic inference. In addition, the present analysis employing COI shares problems inherent to mitochondrial biology, including maternal inheritance, introgression, hybridization, male-biased dispersal patterns, and recent speciation among others [22]; most of these are likely to apply only at the fine-scale level of distinguishing closely-related species. As noted, the indicator method is of general utility and could readily be applied to longer sequences or concatenated multi-gene alignments without substantially increasing computation time, which might address some of these limitations. In this regard, it of interest to compare indicator vector affinities using mitochondrial and nuclear genes in puzzling cases that appear to represent convergent evolution [23].

Although the output is different, it may be revealing to compare the efficiency of the indicator vector approach to that of phylogenetic treebuilding programs. Due to computational demands, data sets in analyses beyond 1000 species are exceptional (e.g., [24]–[26]) and calculation times for larger studies are typically several CPU-months. The largest published phylogenetic tree includes 73,060 eukaryote taxa [1] and took 2.5 months with 16 processors, and the next largest analyzed 13,533 plant taxa [27]. The present study ranks with the largest biodiversity analyses in terms of number of organisms, and is at least two orders of magnitude faster. For example, the case of 12 animal groups deals with almost 17,000 sequences and required times of roughly 10–20 minutes on an ordinary desktop computer. This suggests the potential for analyzing the largest datasets available, including, for example, BOLD (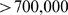 sequences) http://www.barcodinglife.org, NCBI Influenza Virus Resource (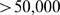 complete genomes) http://www.ncbi.nlm.nih.gov/genomes/FLU/FLU.html, or Los Alamos HIV Sequence Database (http://www.hiv.lanl.gov).

In addition to animals, cytochrome *c* oxidase is present in plants, protozoa, fungi, and some bacteria, which raises the prospect of insight into broad-scale patterns in the genetic structure of biodiversity. Also, the methodology as present here applies to nucleotide sequences of any sort and so might usefully be applied to a variety of questions.

From the point of view of accuracy, density of information and assimilation it would seem compelling that any properly ordered *distance* matrix should be viewed as a Klee diagram. It may be that the focus on evolution and therefore trees impeded this direction. In this connection we note that the distance matrix for a species count of 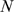 contains 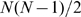 distances and for large 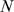 a tree-building algorithm cannot accommodate this number of conditions, and an increasing number of larger and larger errors occur with increasing 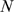. Klee diagrams accurately display distances for any species count.

An important advance in the present treatment derives from the vectorization of nucleotide sequences, (1), which has been accomplished with the exact preservation of Hamming distances. Advantages flow from a vector space framework, an example of which is the optimization procedure leading to the indicator vectors. Another consequence is that bps occupation is rigorously transformed to the probability of occurrence of the four nucleotides, which opens the possibility of introducing information theory into these considerations.
